# Preparation and Characterization of Octenyl Succinic Anhydride Modified Taro Starch Nanoparticles

**DOI:** 10.1371/journal.pone.0150043

**Published:** 2016-02-26

**Authors:** Suisui Jiang, Lei Dai, Yang Qin, Liu Xiong, Qingjie Sun

**Affiliations:** 1 School of Food Science and Engineering, Qingdao Agricultural University, Shandong, China; 2 College of Food Science & Nutritional Engineering, China Agricultural University, Beijing, China; Hanyang University, REPUBLIC OF KOREA

## Abstract

The polar surface and hydrophilicity of starch nanoparticles (SNPs) result in their poor dispersibility in nonpolar solvent and poor compatibility with hydrophobic polymers, which limited the application in hydrophobic system. To improve their hydrophobicity, SNPs prepared through self-assembly of short chain amylose debranched from cooked taro starch, were modified by octenyl succinic anhydride (OSA). Size *via* dynamic light scattering of OSA-SNPs increased compared with SNPs. Fourier transform infrared spectroscopy data indicated the OSA-SNPs had a new absorption peak at 1727 cm^-1^, which was the characteristic peak of carbonyl, indicating the formation of the ester bond. The dispersibility of the modified SNPs in the mixture of water with nonpolar solvent increased with increasing of degree of substitution (DS). OSA-SNPs appear to be a potential agent to stabilize the oil-water systems.

## Introduction

Recently, much attention has been focused on polymeric composite materials filled with nano-sized particles called nanocomposites [1]. Starch nanoparticles (SNPs) are considered one of the most promising polymers for fabrication of polymer nanocomposites and degradable materials, due to their wide availability, biodegradability, impressive mechanical properties, and low permeability [2]. SNPs have been used as good reinforcing fillers in films of thermoplastic starch [3–5], polyurethane [6], polyvinyl alcohol [7], and soybean protein [8]. However, there are abundant hydroxyl groups on the surface of SNPs that decrease the interface compatibility between hydrophilic SNPs fillers and hydrophobic rubber matrices [9]. To overcome this shortcoming, modifications of SNPs by introduction of hydrophobic groups have been reported with various reagents, such as benzyl [10], glacial acetic acid [11], fatty acids [12], and organic solvents [13].

Amphiphilic modification is an effective method of improving the hydrophobicity of starch and has been attracting more researchers’ interest. Amphiphilic polymers have a wide variety of applications, particularly in emulsification, encapsulation, films and coatings, and gel production. Native starch modified by dicarboxylic acid anhydride, containing both hydrophilic and hydrophobic groups, is well known to produce amphiphilic starch and to improve emulsification properties [14–17]. The amphiphilicity of octenyl succinic anhydride (OSA)-modified starch is improved due to the introduction of dual functional hydrophilic and hydrophobic groups [18]. Based on its amphiphilicity, OSA-starch is used as an emulsifier and stabilizer in many food or oil-in-water systems, cosmetics, and pharmaceutical products [19–22]. Furthermore, surface modification through esterification by using OSA significantly increases tensile strength and Young's modulus of starch films [2].

However, amphiphilic modifications of nanoscale starch with OSA are seldom reported. Taro (*Colocasia esculenta*) is widely planted in humid and subhumid tropics and has been reported to have abundant starch with small granules. However, the loss ratio of taro is over 30% during storage. The effective method of transforming taro to taro starch can minimize taro losses and improve the value of taro. A new green method has been established to prepare SNPs through enzymolysis and recrystallization of taro starch [3]. The objective of our study was to create amphiphilic starch nanoparticles (OSA-SNPs) and broaden the potential application as enhanced nanofiller in nanocomposites, or emulsifier and stabilizer in many oil-water systems.

## Materials and Methods

### 2.1 Materials

Laiyang Taro was obtained from Qingdao Academy of Agricultural Sciences, Qingdao, China. Pullulanase (E.C.3.2.1.41, 6000 ASPU/g, 1.15 g/mL [ASPU is defined as the amount of enzyme that liberates 1.0 mg of glucose from starch in 1 min at a pH of 4.4 and 60°C]) was obtained from Novozymes Investment Co. Ltd. (Beijing, China). Octenyl succinic anhydride (OSA) was purchased from Sigma-Aldrich Chemicals (St. Louis, MO). All other reagents used were of analytical grade.

### 2.2 Taro starch nanoparticle preparation

Taro starch (about 14% amylose) was isolated according to the procedures used by Simsek and EI [23]. Briefly, taro tubers were peeled, weighed, sliced, and ground in a commercial blender with triple weight of 0.1% NaOH solution. The slurry was centrifuged at 3500 g for 10 min. The sediment was suspended in water, neutralized with 0.1 N HCl solutions, and centrifuged again. The sediment was then suspended in 0.1 M NaCl solution, and 10% (v/v, based on the volume of 0.1 M NaCl) toluene was added. The solution was stirred overnight at room temperature. After standing for 1 h, the suspension separated into two layers. The bottom layer was centrifuged and the sediment starch was washed with distilled water to completely remove the NaCl, dehydrated with ethanol, and air-dried in an oven at 40°C. The content of the protein and fat in taro starch were 0.82% and 0.40%.

The SNPs were prepared following the method described by Dai et al. [3]. Taro starch slurry (15% w/v in pH 5.0 buffer solutions) was cooked in boiling water and stirred vigorously for 30 min. The cooked starch was cooled to 58°C and pullulanase (30 ASPU/g of dry starch) was added. After 8 h incubation period, the hydrolysate was centrifuged (3000 g, 5 min). The sediment was discarded and the supernatant was heated at 100°C for 10 min to stop the reaction; then, it was cooled to room temperature. The solution was stored at 4°C for 8 h to retrograde. The ensuing nanoparticles were washed several times with distilled water until neutrality was achieved and then freeze dried to obtain SNPs.

### 2.3 Preparation of OSA-SNPs

SNPs (10 g, dry weight) were dispersed in 500 mL 50% ethanol with continuous stirring at 40°C in an ultrasonic cleaner (KQ-500B, Kunshan, China). Then, the SNPs suspension was maintained between pH 8 and 9 by adding NaOH aqueous solution (0.1 mol/L). Different amounts of OSA (50 or 100% based on the weight of the starch) were added (OSA diluted 5 times with absolute alcohol, v/v) slowly over 2 h. After the reaction, the pH was adjusted to 6.8 with 0.1 mol/L HCl solution, the mixture was centrifuged (3000 g, 15 min), washed three times with distilled water and twice with 70% aqueous alcohol, and freeze dried. At this point, two samples, 50% OSA-SNPs and 100% OSA-SNPs, were obtained respectively.

### 2.4 Dynamic light scattering (DLS)

The average size and size distribution of the nanoparticles were determined by dynamic light scattering (DLS) using a Malvern Zetasizer Nano (Malvern Instruments Ltd., UK) equipped with a He-Ne laser (0.4 mW, 633 nm) and a temperature-controlled cell holder. The measurements were performed in samples diluted in Milli-Q water and analyzed at 25°C. The samples were sonicated before testing. The mean intensity-weighted diameter was recorded.

### 2.5 Transmission electron microscopy (TEM)

TEM images of SNPs and OSA–SNPs were taken with a 7650 transmission electron microscope (Hitachi, Tokyo, Japan) with an acceleration voltage of 80 kV. SNPs and OSA-SNPs suspensions (0.01%, w/v) were sonicated for 1 min. A tiny drop of suspension was deposited on a carbon coated copper grid containing 400 meshes.

### 2.6 Wettability tests

The wettability test is a useful method for validating the characterization of SNPs polarity. For these tests, SNPs and OSA-SNPs were mixed with two immiscible solvents (5 mL each), distilled water (*d* = 1g cm^-3^), chloroform (*d* = 1.47 g cm^-3^), dichloromethane (*d* = 1.335 g cm^-3^) and toluene (*d* = 0.866 g cm^-3^) which have different polarities and densities. Pictures were taken to illustrate the solubility of SNPs after modification in water/nonpolar solvents.

### 2.7 Fourier transform infrared spectroscopy (FTIR)

Fourier transform infrared spectra were recorded using a Jasco FTIR 4100 spectrometer (Jasco Inc., Easton, MD, USA). SNPs and OSA-SNPs were collected using the KBr pellet method. A total of 32 scans were obtained and the resolution was 4 cm^-1^. The wavelength region was between 4000 and 400 cm^-1^. All spectra were baseline corrected and normalized through setting the maximum transmittance to 100%.

### 2.8 Determination of the degree of substitution

Degree of substitution (DS) is the average number of hydroxyl groups substituted per glucose unit. The DS of the OSA-SNPs was determined using the titration method [24]. A known weight of the sample was dissolved in 10 mL of DMSO by heating (70°C, 10 min). After cooling, 5–6 drops of phenolphthalein indicator were added. This solution was titrated against 0.05 M standard NaOH solution until a permanent pale pink color was seen. The DS was calculated by using the following equation:

$$DS=\frac{0.162\times V\times M}{1000\times (W-266.38)\times V\times M}$$ (1)

where: V is the volume of NaOH solution used during titration; M is the molarity of the NaOH solution and W is the weight of sample analyzed.

### 2.9 X-ray diffraction

The X-ray patterns of SNPs and OSA- SNPs were analyzed using an X-ray diffractometer (AXS D8 ADVANCE; Bruker, Karlsruhe, Germany) with Cu Kα radiation at a voltage of 40 kV and 30 mA. The samples were scanned with a *2*θ angle range of 3–40°. Samples crystallinity was determined by plotting the peaks baseline on the diffractogram and calculating the area using the software spectrum viewer (Version 2.6) according to the method described by Jivan, Madadlou, and Yarmand [25]. The area above and under the curve corresponded to crystalline domains and amorphous regions, respectively. The ratio of upper area to total area was taken as relative crystallinity:

Relative crystallinity(%)=(Area under the peaks/Total curve area)×100 (2)

### 2.10 Thermogravimetric analysis (TGA)

TGA was carried out using the synchronous thermal analysis (STA449C/4/G, Netzsch, Selb, Germany). Samples weighing 3–6 mg were heated from 25°C to 400°C at a heating rate of 10°C min^−1^ in a dynamic atmosphere of synthetic air (80% N_2_ and 20% O_2_) and a flow rate of 100 mL/min.

### 2.11 Statistical analysis

Each measurement was carried out using at least three fresh, independently prepared samples. The results were reported as the mean value and standard deviation. The data were subjected to analysis of variance (ANOVA) using the SPSS V.17 statistical software package (SPSS Inc., Chicago, IL). Duncan’s multiple range test was also applied to determine the difference of means from ANOVA, using a significance test level of 5% (p < 0.05).

## Results and Discussion

### 3.1 The size distribution of SNPs and OSA-SNPs

**Fig 1:**
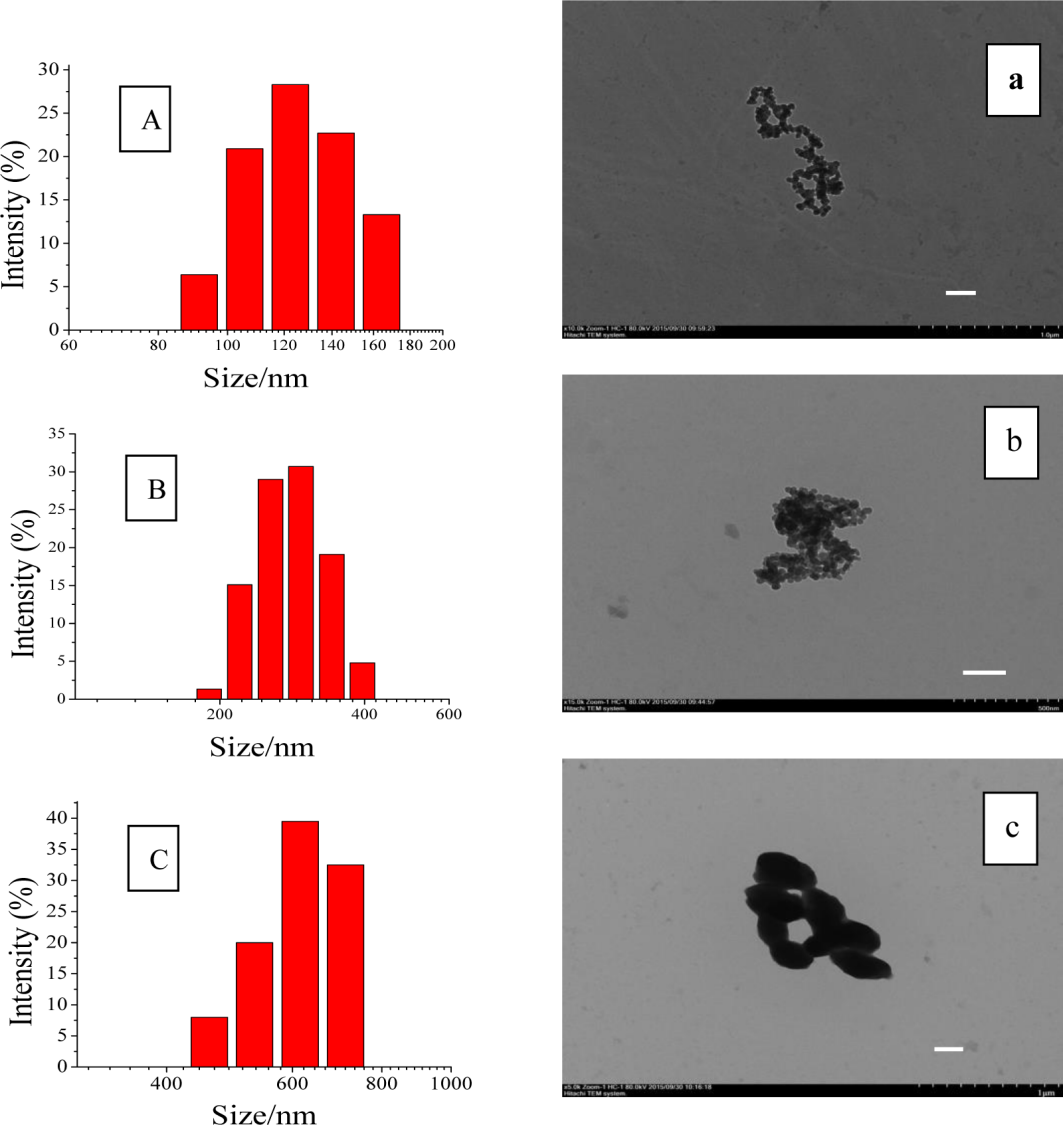
Particle size distribution and transmission electron microscopic images of starch nanoparticles (A, a), octenyl succinic anhydride modified starch nanoparticles with degree of substitution of 0.024 (B, b) and 0.036 (C, c). The scale bars represent a length of 200 nm.

OSA-SNPs with different DS were prepared by varying the addition amount of OSA. The DS were 0.024 and 0.036 respectively while the 50% and 100% of OSA (based on the weight of the starch) were added (Table 1). The shape of the SNPs and OSA-SNPs was oval or round (Fig 1A–1C). The diameters of SNPs, OSA-SNPs with DS of 0.024 and 0.036 in TEM images were approximately 40–60 nm, 60–80 nm, and 150–200 nm, respectively. The DLS results showed that SNPs (Fig 1A) gave relatively small size distribution, ranging from 90 nm to 160 nm, with an average diameter of 132 nm. The OSA-SNPs with the DS of 0.024 and 0.036 possessed average diameter of 295 nm and 493 nm, respectively (Fig 1B and 1C). The diameters of nanoparticles observed by DLS were generally larger than that obtained by TEM. One reason was that the diameter of nanoparticles obtained by DLS reflected the hydrodynamic diameter of nanoparticles swelled in aqueous solution, while that observed by TEM was the diameter of dried nanoparticles. Another reason was probably due to the self-assembly of amphiphilic particles. In water medium, the hydrophobic alkenyl groups of OSA among adjacent nanoparticles combined to form a hydrophobic microenvironment, and the hydroxyl groups on the surface of the OSA-SNPs were in extended form exhibiting hydrophilic character. Thus, adjacent nanoparticles gathered to form aggregate by hydrophobic interactions, this can be seen from the OSA-SNPs with the DS of 0.024 in Fig 1B. While the DS was 0.036, there were more hydrophobic groups and increased hydrophobic force, leading to the formation of larger nanoparticles (Fig 1C). Therefore, the OSA-SNPs with a higher DS also had a larger size. Likewise, previous study has indicated that as the hydrophobic groups increased, the size of amphiphilic nanoparticles increased [26].

**Table 1:** Relative crystallinity (%) and degree of substitution (DS) of octenyl succinic anhydride modified taro starch nanoparticles (OSA-SNPs).

Sample	Crystallinity (%)	DS
SNPs	57.78±0.70^a^	0
OSA-SNPs (50%)	51.64±0.30^b^	0.024±0.03^b^
OSA-SNPs (100%)	48.16±0.50^c^	0.036±0.02^a^

Values are means±standard deviations of three replications. Different letters (a, b, c) in the same column represent significant differences (p<0.05).

OSA-SNPs (50%, 100%): SNPs modified with 50% and 100% OSA (based on the weight of starch).

### 3.2 Wettability tests of SNPs and OSA-SNPs

**Fig 2:**
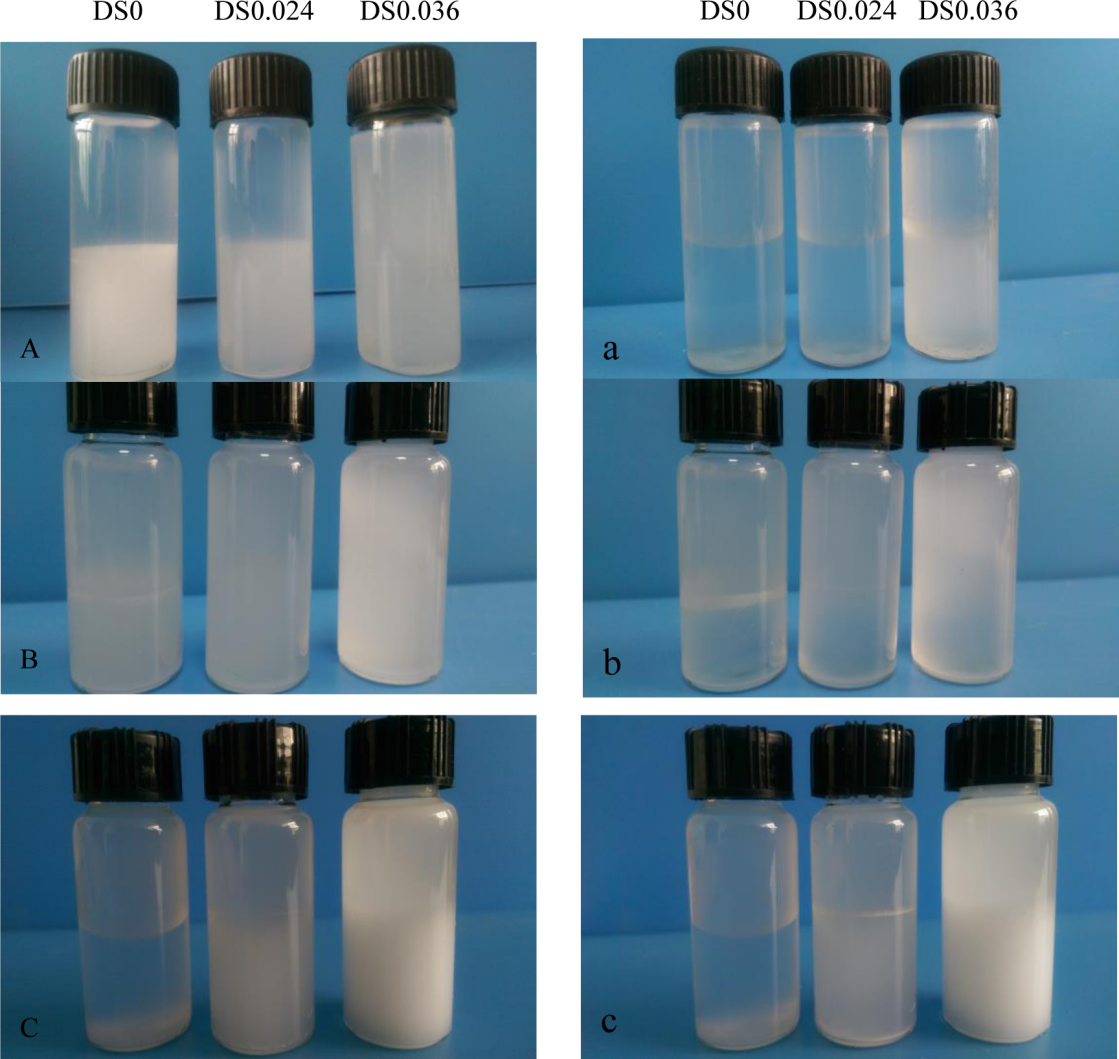
Photographs of dispersibility of starch nanoparticles and octenyl succinic anhydride modified starch nanoparticles with different degree of substitution (DS) in water/ toluene was taken after 30 s (A) and 2 h (a), in water/ dichloromethane was taken after 30 s (B) and 2 h (b), in water/ chloroform was taken after 30 s (C) and 2 h (c).

The introduction of hydrophobic alkenyl groups into the molecular structure of SNPs is effective to alter their surface properties. In particular, the wettability of SNPs was tested *via* ultrasound treatment after adding SNPs to different solvent systems (water/toluene = 1:1, v/v; water/ dichloromethane = 1:1, v/v; water/chloroform = 1:1, v/v). The polarities of water, toluene, dichloromethane, and chloroform are 10.2, 2.4, 3.4, and 4.4, respectively. As shown in Fig 2A, SNPs remained in water phase due to hydrophilic character of abundant hydroxyl groups. In contrast, OSA-SNPs could also migrate to toluene phase, suggesting amphiphilic character after modification of SNPs by OSA. When the DS increased, more and more OSA-SNPs migrated toward toluene. Higher DS of OSA-SNPs implied that SNPs contained more carbonyl group or alkenyl groups, which resulted in lower polar nature of the modified SNPs. Similar trend was also observed in dichloromethane and chloroform solvent (Fig 2B and 2C). The OSA-SNPs with DS of both 0.024 and 0.036 exhibited better wettability in dichloromethane than those in toluene and chloroform (Fig 2A, 2B and 2C). These results reflected that OSA-SNPs had amphiphilic character due to the addition of dual functional hydrophilic and hydrophobic groups. When OSA-SNPs was in water medium, the hydrophilic hydroxyl groups of SNPs was in stretched in water exhibiting hydrophilic character and the hydrophobic alkenyl group of the OSA was rolled up. In contrast, in toluene, dichloromethane and chloroform solvent, the hydrophobic alkenyl group of the SNPs was in stretched form showing hydrophobic character [9]. Similarly, Ren et al. [27] found that more OSA-modified SNCs could migrate into toluene, indicating the OSA-modified SNCs had higher hydrophobicity after modification.

The change of solvent system stability being stored at room temperature was observed in Fig 2. The suspensions of OSA-SNPs dispersed uniformly in the mixture of water with nonpolar solvent (water/chloroform = 1:1, v/v; water/ dichloromethane = 1:1, v/v; water/toluene = 1:1, v/v) after 30 s of storage. After 2 h of storage, the creaming of SNPs and OSA-SNPs with DS of 0.24 were serious and OSA-SNPs with DS of 0.36 were slight. The result indicated that OSA-SNPs suspension with DS of 0.36 was more stable than both of the SNPs suspension and OSA-SNPs suspension with DS of 0.24. This was because that a higher DS of OSA-SNPs meant that the particles contain more octenyl succinate groups, which had a strong ability to reduce interfacial tension. They could arrange themselves compactly at the oil/water interface to form a highly viscous interfacial film. Thus solvent system stability was better with higher DS OSA-SNPs.

### 3.3 Fourier transform infrared spectroscopy (FTIR) analysis

**Fig 3:**
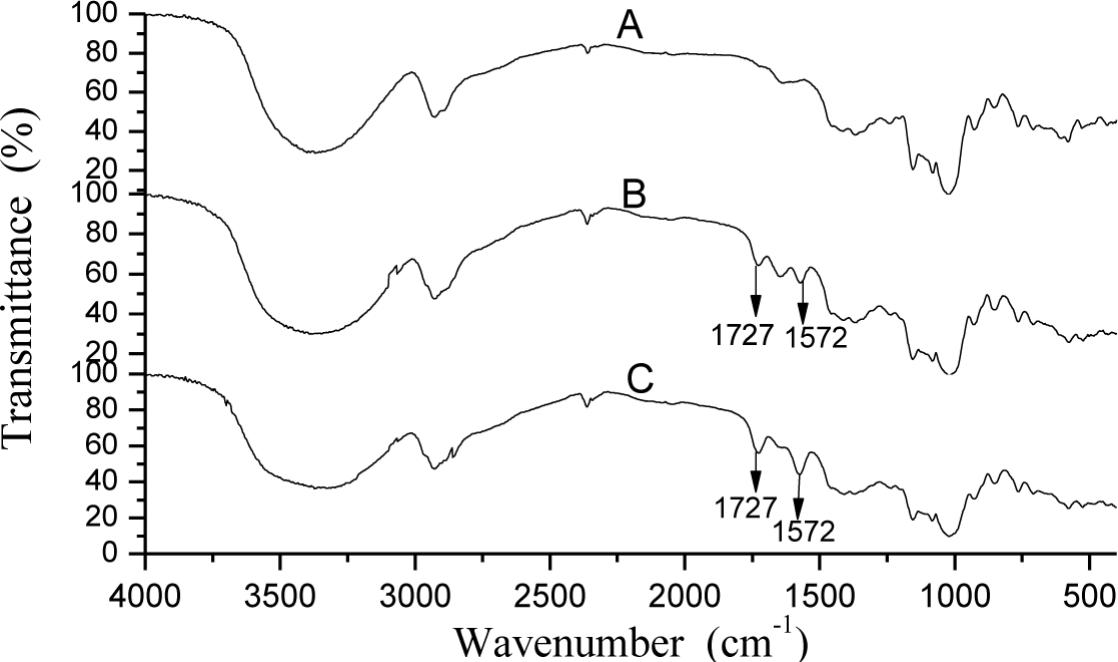
FTIR of taro starch nanoparticles (A), octenyl succinic anhydride modified taro starch nanoparticles with degree of substitution of 0.024 (B) and 0.036 (C).

To determine whether the esterification successfully formed, the FTIR spectra of SNPs and OSA-SNPs were obtained (Fig 3). The peaks appearing at 3000–3800 cm^-1^ in the spectrum were due to hydrogen-bonded hydroxyl groups (O-H). The band at 2927 cm^−1^ was characteristic of C–H stretching associated with anhydroglucose of SNPs and OSA-SNPs and the peak near 850 cm^−1^ corresponded to the C–H deformations. Another two characteristic peaks at 1153 and 924 cm^−1^ were attributed to C-O bond stretching. The absorption at about 1644 cm^−1^ is due to residual bound water in the samples. Compared with the SNPs curve, two new absorption bands at 1727 and 1572 cm^−1^ appeared in OSA-SNPs curves [16]. The peaks at 1727 cm^−1^ were the stretching vibration of C = O [28]. This indicated the formation of ester carbonyl groups in the OSA-SNPs [29–30]. The new peak at 1572 cm^−1^ was due to the asymmetric stretching vibration of carboxylate RCOO− [30]. As presented in Table 1, the DS of was 0.024 and 0.036, respectively. The intensities of OSA-SNP with DS of 0.036 absorption bands at 1727 and 1572 cm^−1^ were higher than those of 0.024 (Fig 3), which showed that the intensities of two peaks (1727 and 1572 cm^−1^) increased with the DS. The result was consistent with previous reports [28–29, 31].

### 3.4 X-ray diffraction pattern analysis

**Fig 4:**
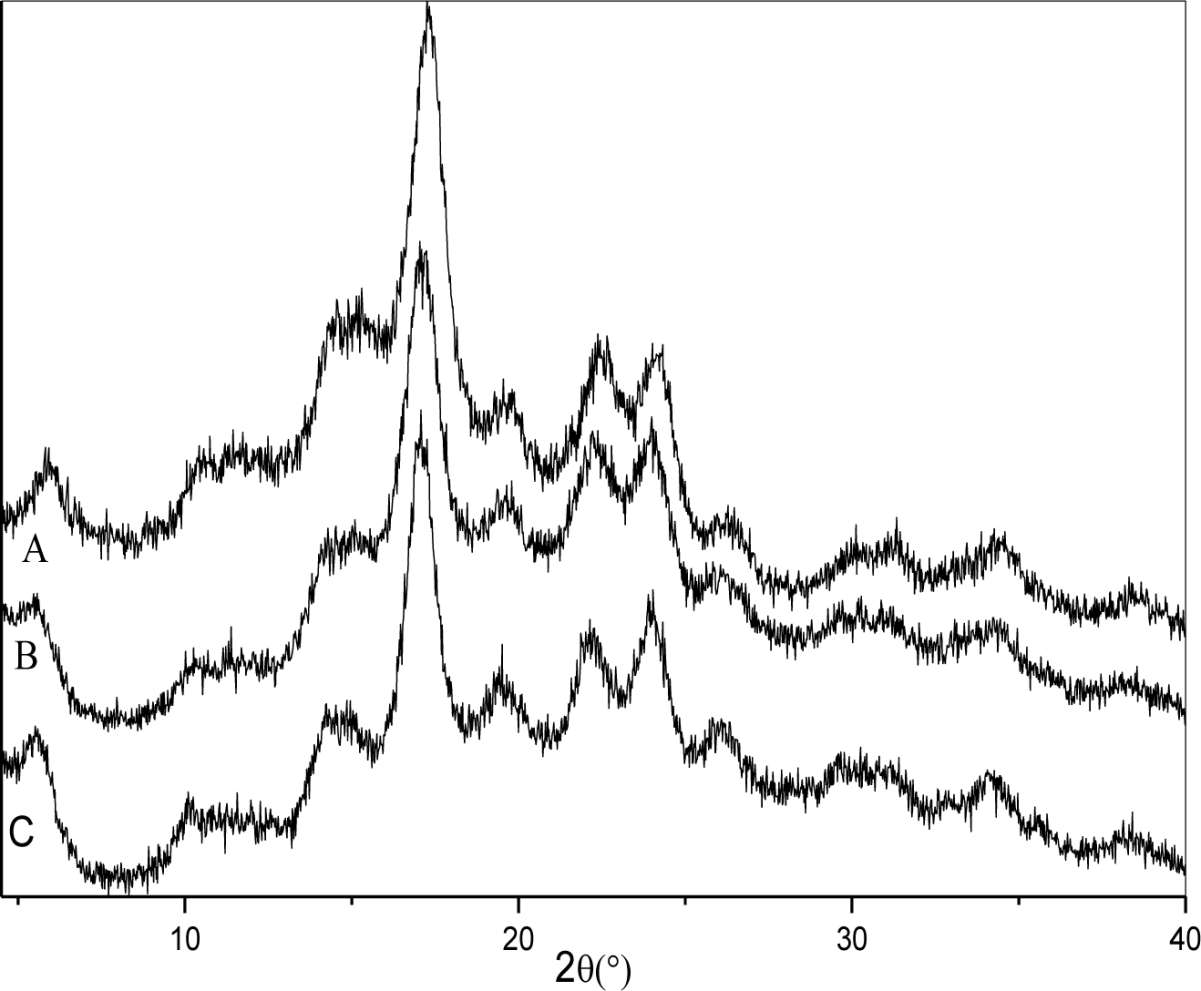
X-ray diffraction patterns of taro starch nanoparticles (A), octenyl succinic anhydride modified taro starch nanoparticles with degree of substitution of 0.024 (B) and 0.036 (C).

The X-ray diffraction patterns of SNPs and OSA-SNPs are shown in Fig 4 and the crystallinity is shown in Table 1. SNPs exhibited diffraction peaks at Bragg angles (2*θ*) of 5.71°, 14.51°, 17.09°, 19.52°, 22.40°, and 24.01°, representing the B-type crystalline structure. This result was in agreement with the report of Dai et al. [3]. In their study, SNPs prepared from taro starch after gelatinization, debranching, and retrogradation also showed a B-type X-ray patterns. The X-ray diffraction patterns of SNPs did not change after OSA modification. The OSA-SNPs also exhibited typical B-type diffraction patterns. This result suggested that OSA modification did not change the crystalline patterns of the SNPs, indicating that the esterification reaction mainly occurred in amorphous regions in the SNPs. However, part of the crystalline structure of the SNPs was damaged after OSA modification, because the intensity of characteristic X-ray peaks was lower in OSA-SNPs than in SNPs. As shown in Table 1, the crystallinity of the SNPs (57.78%) was higher than that of the OSA-SNPs with DS of 0.024 (51.64%) and OSA-SNPs with DS of 0.036 (48.16%). In the alkaline systems, NaOH can destroy the crystalline structure of SNPs and make it easier for OSA to react with the SNPs. This result was in accordance with the report of He et al. [18], who indicated that esterification did not change the crystalline patterns of rice starch. Ren et al. [23] also reported that the crystalline structure of starch nanocrystals prepared by acid hydrolysis was preserved after the OSA modifications. Since the crystalline structure of OSA-SNPs was not disrupted and hydrophobicity of SNPs was significantly enhanced, the modified SNPs can be used as reinforcements to prepare hydrophobic polymer based nanocomposites and those needing non-polar solvents for casting fabrication.

### 3.5 Thermogravimetric analysis

**Fig 5:**
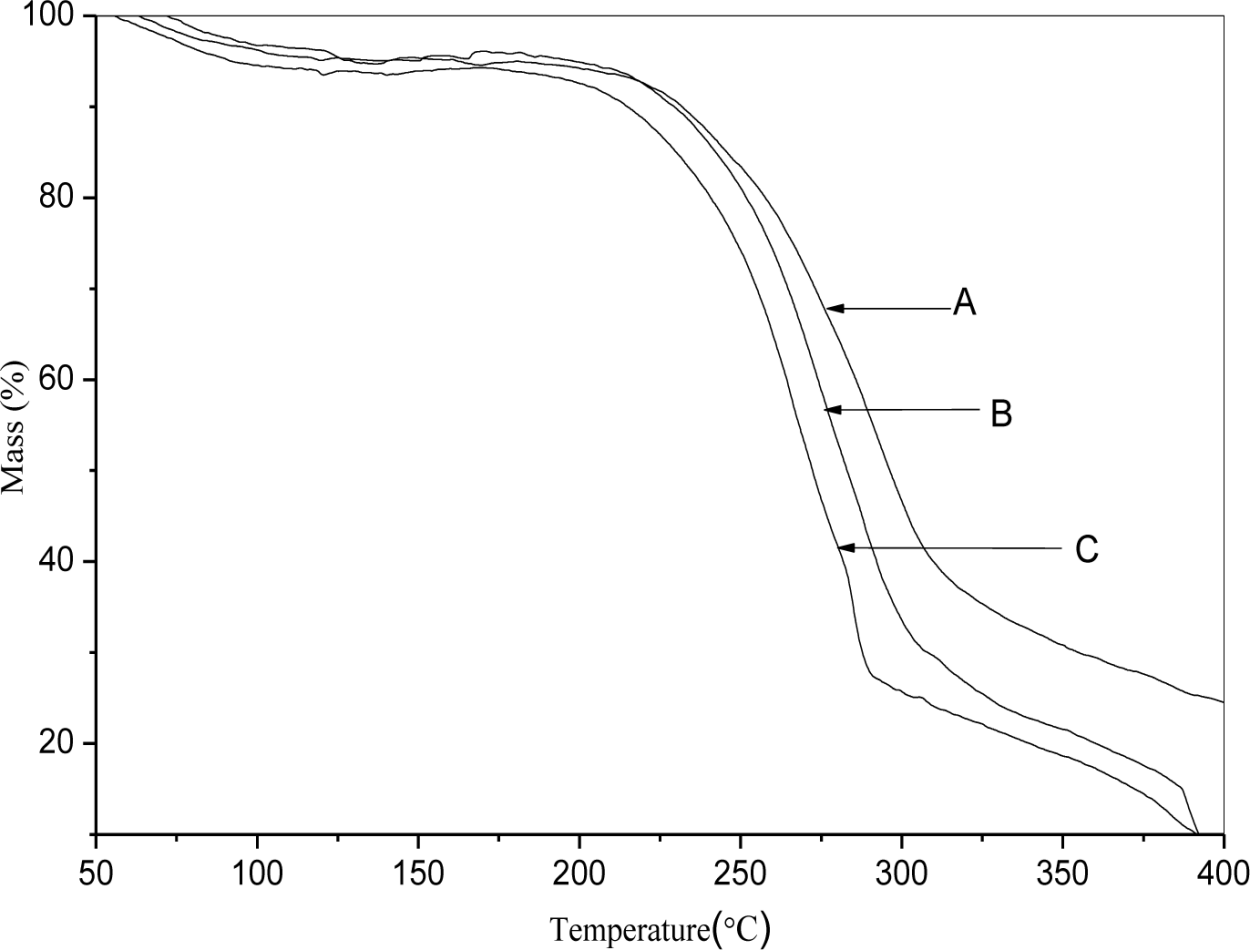
Thermal gravimetric analysis thermograms of taro starch nanoparticles (A), octenyl succinic anhydride modified taro starch nanoparticles with degree of substitution of 0.024 (B) and 0.036 (C).

TGA is a useful technique to weight change of SNPs as a function of temperature, and to assess their thermal stability. TGA results are illustrated in Fig 5, three distinct regions can be seen in these thermogravimetric curves. The initial weight loss (before 100°C) was generally due to evaporation of water. The second stage was the main thermal decomposition zone of SNPs, and the final stage was generally carbonization.

For SNPs, the main mass loss step occurred at 230–320°C, and the temperature at the maximum thermal decomposition rate was about 296°C. However, the depolymerization of the OSA-SNPs started earlier than that of the SNPs. The main mass loss step for OSA-SNPs with DS of 0.024 and OSA-SNPs with DS of 0.036 occurred at the 220–315°C and 200–285°C, respectively. Thus, the thermal stability of the SNPs decreased after OSA modification. This was ascribed to the disruption of part of the crystalline structure of the SNPs after modification with OSA. The hydrophobic groups of OSA were introduced in the molecular chain of SNPs, altering the SNPs’ hydrophobicity and hydrogen bonding, weakening the interactions between SNPs, and resulting in lower thermal stability. Similar results have been reported by Bao et al. [32], where the OSA weakened the internal hydrogen bonding of rice, wheat, and potato starches. The temperature at the maximum thermal decomposition rate of the OSA-SNPs with DS of 0.036 (270°C) was much lower than that of the OSA-SNPs with DS of 0.024 (283°C). This was because the internal hydrogen bonding of OSA-SNPs with DS of 0.036 was more weakened.

## Conclusion

The properties of SNPs are greatly affected by the properties of introduced groups and DS. It has been demonstrated that OSA-SNPs had amphipathy and dispersed well in the mixture of water with nonpolar solvent. The amphiphilicity of OSA-SNPs increased with the increase of DS. FTIR showed the characteristic vibration of the ester carbonyl groups in the OSA-SNPs at 1727 cm^-1^. X-ray diffraction result confirmed that the esterification reaction mainly occurred in amorphous regions of SNPs. The presented work provides a route to improve dispersion of SNPs in polar/nonpolar solvent and broaden the potential application as a stabilizing agent in many oil-water systems.
